# Effects of adding video feedback to emotionally focused therapy supervision: A concurrent multiple‐baseline across subjects design

**DOI:** 10.1111/jmft.12586

**Published:** 2022-03-07

**Authors:** Andrea K. Wittenborn, Sailaja Subramaniam, Preston C. Morgan, Chi‐Fang Tseng

**Affiliations:** ^1^ Department of Human Development and Family Studies Michigan State University East Lansing Michigan USA; ^2^ Department of Psychiatry and Behavioral Medicine Michigan State University Grand Rapids Michigan USA

**Keywords:** emotionally focused therapy, multiple‐baseline design, supervision, video feedback

## Abstract

Emotionally focused therapy (EFT) is an empirically supported intervention for relationship distress with an established model of supervision. This study examined whether incorporating video feedback (VF) software into EFT supervision would improve therapists' level of development compared to traditional EFT supervision in a university training clinic. A concurrent multiple‐baseline across subjects design, along with a thematic analysis of qualitative data, were used in this proof‐of‐concept study of the new supervision component. Overall, quantitative findings suggested that using VF in EFT supervision resulted in some improvement to therapists' development, while qualitative findings showed that all participants supported the incorporation of VF into EFT supervision. Future research on VF is needed to provide additional insight into the use of video review supervision.

## INTRODUCTION

Emotionally focused therapy (EFT) is an empirically supported intervention for relationship distress that is informed by attachment theory and draws on experiential and emotional processes. Therapists learn to deliver EFT by facilitating therapeutic change through a three‐stage process, which involves deescalating negative cycles of interactions, restructuring interactions, and consolidating change (Johnson, 2019). EFT therapists learn to leverage emotion among partners in session to restructure interactions and increase attachment security. The EFT supervision model provides a framework for training therapists to develop skills in the delivery of EFT (Palmer‐Olsen et al., 2011). Effective supervision is premised upon the clarity, specificity, and timing of the feedback provided by the supervisor. Common supervision formats, such as live and case report methods, can present some limitations with respect to an EFT supervisor's capacity to provide timely feedback that is embedded within the emotional context of a session. Common supervision formats may also create barriers to assisting therapists in deepening emotional experiencing among clients as it removes the supervisor and supervisee from the emotional experience occurring in session. Overcoming these limitations to common supervision formats is critical since facilitating deep emotional experiencing among clients is associated with better clinical outcomes (e.g., Castonguay et al., 1996; Furrow et al., 2012; Peluso & Freund, 2018; Watson & Bedard, 2006).

When EFT therapists access and process clients' intense emotions in session, clients are able to engage in corrective emotional experiences and complete key therapeutic change events that lead to better clinical outcomes for couples (Bradley & Furrow, 2004; Furrow et al., 2012). Effectively guiding clients into deep emotional experiencing in key EFT change events transpires through therapists' use of evocative interventions such as heightening, evocative responding, and empathic conjecture (Bradley & Furrow, 2004). While deep emotional experiencing, including softening events, is a well‐known indicator of effective EFT, it is considered the most difficult skill for therapists to learn (Bradley & Furrow, 2007; Johnson & Greenberg, 2007). The importance of developing these skills and the limitations posed by common supervision formats have led to calls for new approaches to enhance the supervisory process, including the integration of new technologies (Champe & Kleist, 2003).

Video feedback (VF) is a technology‐assisted approach that has been used to enhance professional training in a range of applications such as surgery, nursing, and education (e.g., Augestad et al., 2020; Noordman et al., 2014). VF provides the capacity to digitally make notations and flag important moments directly on a recording during live or video supervision. In live or video supervision, supervisory feedback can be recorded digitally and a supervisee can playback the brief segments of a session that were electronically flagged by the supervisor to more efficiently view the focal therapy segment and review the supervisor's observations as it pertains to that specific moment in therapy. This technology‐assisted approach offers several practical benefits. First, VF can enhance supervision by highlighting supervisor‐identified key moments for review and creating opportunities to provide richer feedback embedded within the context of the session. Returning to the key moment flagged for review offers an opportunity to heighten therapists' awareness of their own practice, challenge assumptions, and engage in reflective practice in a safe setting. Second, VF may improve the efficiency of supervision in some settings. A common complaint about video supervision is that it is time‐consuming to review videos of full‐length therapy sessions and identify key moments to review in consultation with a supervisor. Therapists‐in‐training are often responsible for locating key moments for video review and the video segments they select often do not align with supervisors' own recommendations of key moments to review. VF increases the efficiency of this process by enabling supervisors to digitally flag key therapy moments during live or video supervision that are then easily accessible within the video review user interface. It also enables supervisors to rely on their expertise to identify segments that display the strongest need for improvement rather than asking the therapist‐in‐training to assume this responsibility.

VF is also aligned with the specific goals and tasks of the EFT model of supervision. EFT supervision is isomorphic to the practice of EFT (Palmer‐Olsen et al., 2011) and often requires therapists to process their own emotional experiences of clients or therapeutic events. Therapists‐in‐training may be overcome by their own emotional processes in session and VF can assist in the identification of key therapeutic moments to facilitate an experiential learning opportunity in which therapists can process their own emotional experiences within an appropriate and safe environment outside of the session. VF can also be used to support therapists in identifying clients' emotional processes, attachment needs, and interactional cycles. Relevant information revealed by clients in session can be flagged and reviewed for additional reflection in supervision as needed. Further, hot keys or keyboard shortcuts can be preprogrammed in VF that enable supervisors to type one letter on a keyboard to flag a segment of the session and code it as a specific technique, common therapeutic process, or otherwise in need of review.

### Current study

Prior research on clinical supervision has been relatively sparse (Bambling et al., 2006; Bartle‐Haring et al., 2009). The present study aimed to expand the empirical literature on clinical supervision of EFT to explore the addition of VF. VF was recently integrated into a university training setting for therapists learning EFT and this study formally evaluated the use of VF. Specifically, this study assessed whether adding VF to EFT supervision improved therapists' level of development. A concurrent multiple‐baseline across subjects design was used in this proof‐of‐concept study of the new supervision component.

## METHOD

This section provides an overview of the study protocol. This study was approved by the Institutional Review Board at Michigan State University.

### Design

A concurrent multiple‐baseline across subjects design was used to evaluate whether adding VF to EFT supervision improved therapists' level of development. Multiple‐baseline designs are especially useful in evaluating the effects of adjusting a multicomponent intervention, including the addition of an intervention component, on a dependent variable (Kratochwill et al., 2010). Multiple‐baseline designs have the potential to demonstrate an inferred causal relation between the dependent variable and the intervention. In this design, intervention effects are demonstrated by introducing the additional intervention component to different participants at different points in time (Kazdin, 2020). Participants' assessments are expected to develop a stable or predictable pattern during the baseline phase before the introduction of the additional intervention component. Changes in the trajectories of participant assessments following the introduction of the additional intervention component can be attributed to the intervention. In this design, a participant essentially serves as their own control condition as the design allows comparisons of participant assessments before and during the intervention. Another benefit of multiple‐baseline designs is that they can accommodate small sample sizes. The Institute of Educational Science's What Works Clearinghouse standards for single‐case data analysis (Kratochwill et al., 2010) recommends a sample of at least three participants but clarifies that four or more participants are more desirable due to the increase in power when a statistical test is used. Further, Lanovaz and Turgeon's (2020) analysis suggests that showing a clear change (i.e., stable trend in visual analysis) on at least two of three participants results in acceptable power (>0.80) and Type I error (<0.05). Internal validity was sought in the current study by testing the intervention across four participants who were randomized to different baseline lengths (Kratochwill et al., 2010).

### Procedures

Participants were recruited from a couple and family therapy (CFT) doctoral‐level practicum focused on learning EFT. Therapist interns were each asked to select one focal couple with whom to deliver EFT and receive EFT supervision on throughout the study. The clinical supervisor in the study was a licensed marriage and family therapist with expertise in EFT. Participants were randomly assigned to different baseline lengths to control for potential biases. During the baseline phase, participants were supervised using the EFT model of supervision with the same couple following the plan–observe–feedback format; in this format, therapist interns received case report and live supervision. During the intervention phase of the study, the model and format of supervision continued, except that VF supervision was added. For the VF supervision component, therapist interns were asked to watch specific moments in their therapy sessions that were electronically flagged during live supervision by the supervisor and read the supervisor's observations pertaining to the video segments. The baseline phase of the study coincided with the start of a semester‐long practicum course that aimed to train students in EFT.

Participants were assessed after each session with their focal couple throughout the baseline and intervention phases. The primary outcome in this study was therapist development measured by the Supervisee Level Questionnaire—Revised (SLQ‐R; McNeill et al., 1992). In addition, qualitative survey responses were collected at the end of the intervention phase and 4 months after the intervention ended to assess for social validity (e.g., acceptability and satisfaction). The qualitative items measured participants' perceptions of the appropriateness of VF and their satisfaction with it.

### Participants

Participants included two doctoral‐level therapist interns and two master's‐level social work students practicing CFT in a university‐based clinic. Three participants identified as female and one identified as male. Participants reported an age range of 26–32 years. Each participant was randomized to a predetermined number of days in the baseline phase.

### Supervision intervention

This study evaluated the addition of VF to EFT supervision using the plan–observe–feedback format. More specifically, the supervision provided during the baseline phase involved the supervisor and CFT intern meeting to plan for the session, the supervisor observing the live session, and the supervisor providing verbal feedback to the CFT intern during or after the session ended. VF was then added during the intervention phase. VF is a technology‐assisted approach to supervision in which the supervisor can electronically flag important moments in the therapy session and record digital feedback directly onto a live or video‐recorded session. This study used Morae software, though other software packages may offer similar features (e.g., Lookback, Video Review, and Vimeo). This software affords the supervisee the opportunity to playback important segments of the therapy session and return to the emotional field of the moment while reflecting on the supervisory feedback. In addition to providing general feedback, the supervisors could flag video segments according to specific preprogrammed hot keys or keyboard shortcuts in which supervisors could efficiently type one letter on the keyboard to code a segment in a specified way (e.g., as focusing on a specific therapeutic process). The preprogrammed markers in this study included: (a) alliance, (b) interactional pattern, (c) reframe, (d) pursuer softening, (e) withdrawer re‐engagement, (f) enactment, (g) blocking, (h) emotional experiencing, (i) consider being more active, (j) client showing growth, (k) adverse event discussed, and (l) bring to supervision.

The therapist‐in‐training was instructed to watch video segments of their sessions that were electronically flagged by the supervisor and read the supervisor's feedback before the next planning session. Therapists‐in‐training could also filter the electronic feedback based on the specific marker types to efficiently review, for example, all video segments and supervisory feedback that the supervisor identified as needing to discuss or those related to opportunities for or effective facilitation of deep emotional experiencing. Pooling the supervisory feedback on specific markers over the course of therapy with a couple offered opportunities to highlight patterns in practice and reflect on ways to shift patterns that were not conducive to facilitating change.

### Measures

Participants completed the SLQ‐R (McNeill et al., 1992) after each session with their focal couple. The SLQ‐R is a 30‐item self‐report measure of therapists' level of development. An early analysis of the psychometrics of the SLQ‐R suggested a three‐factor structure, including a total score and three subscale scores (i.e., self and other awareness, motivation, and dependency–autonomy). However, a more recent analysis of the SLQ‐R psychometrics conducted with a larger sample size found that only 25 of the initial 30 items should be used to calculate a total score and only identified two subscales, professional self‐confidence (i.e., 14 items that describe positive self‐evaluations in understanding and treating clients) and professional insecurity (i.e., 11 items that describe doubts and worries about their role as a therapist; Junga et al., 2019). The two‐factor structure resulted in improved psychometrics; therefore, Junga et al.'s (2019) recommendations were followed in the current study. Therapist interns rated items from 1 (*Never*) to 7 (*Always*), with a possible range of 25–175 for the total score. We reverse coded the professional insecurity subscale items such that higher scores always reflected more desireable levels of developmentparti; we refer to this subscale as professional security in this article for ease of interpretation. The SLQ‐R has good internal consistency (*α* = 0.92; Junga et al., 2019), and Cronbach's alpha in this study was 0.80.

Open‐ended qualitative survey questions were also used to measure participants' satisfaction with and acceptance of the supervision intervention. At the end of the intervention phase, participants were asked about their experiences of and satisfaction with VF. In the 4‐month follow‐up interview, we asked participants the following: (1) How has discontinuing VF affected your ability to integrate lessons learned from therapy and advance your EFT skills? (2) How has your progress in EFT been affected by being involved in this study? and (3) Would you recommend the use of VF in clinical training programs?

### Data analysis

#### Multiple‐baseline analysis

A combination of visual and statistical methods was used to evaluate the supervision intervention. In the current study, the data were visually inspected for consistent trends within and between each phase, including limited variability within each phase (Kratochwill et al., 2010). More specifically, the visual analyses were guided by the Institute of Educational Science's What Works Clearinghouse (WWC) standards for single‐case data analysis (Kratochwill et al., 2010), which involves a four‐step process: (1) demonstrate a predictable baseline pattern, (2) assess whether the data are sufficient to demonstrate a consistent pattern within each phase, (3) assess whether the introduction of the intervention is associated with a change in the dependent variable, and (4) evaluate whether there are demonstrations of an effect. Further, six features were used to assess patterns within the data: (1) level, (2) trend, (3) variability, (4) immediacy of effect, (5) overlap, and (6) consistency of patterns. The WWC standards suggest that strong evidence must have three participants and moderate evidence must have two demonstrations of effect; however, this standard was not grounded in empirical evidence. In turn, Lanovaz and Turgeon (2020) completed an empirical analysis that showed the standard was overly stringent. Instead, their empirically‐informed guidelines for Type I error rate and power based on the number of tiers in the study (i.e., defined in our analysis as participants) suggest that a clear change (i.e., visual analysis showing stable trend) for at least two of three participants would result in acceptable power (>0.80) and Type I error (<0.05).

The standards for single‐case data analysis recommend a minimum of three data points per phase to meet standards with reservations and at least six data points per phase to meet standards without reservations (Kratochwill et al., 2010). In the current study, one participant did not meet the minimum threshold in one phase (i.e., one participant had two data points in the intervention phase) due to missing data and was removed from the quantitative analyses.

In addition to visual inspection, effect size estimates were calculated for each participant using the tau‐*U*. The tau‐*U* is a nonparametric estimate of effect size that examines the overlap between the baseline and intervention phases (Parker et al., 2011). Estimating effect sizes in single‐case design analyses provide an additional method for assessing whether there was a treatment effect beyond the response expected from the baseline trend (Tarlow, 2017). Another type of effect size estimate called the baseline‐corrected tau single‐case statistic (BC‐tau) can improve upon the tau‐*U* in some cases; however, BC‐tau can overestimate baseline correction effects that have five or less baseline time points (Tarlow, 2017), which was the case for participants 1 and 2 in this study. Hence, we used the tau‐*U* because it provided more conservative estimates of baseline corrections given the number of baseline data points in the current study. To calculate the effect size, data from the baseline phase was evaluated to determine if a statistically significant baseline trend was present. If a baseline trend was detected, the data were corrected. The next step involved calculating the effect size by comparing data from the baseline and intervention phases. In this study, an online tau‐*U* calculator was used to estimate the effect sizes (Vannest et al., 2016). A positive, significant effect size indicates that participants improved during the intervention phase, while a negative or nonsignificant effect size suggests participants did not experience significant improvement when VF was added to supervision.

#### Thematic analysis

We conducted a thematic analysis based on Braun and Clarke's (2006) recommendations to further assess the social validity of the multiple‐baseline analysis. The postintervention and 4‐month follow‐up qualitative data were analyzed separately using an identical process. Data from all four participants were included. To complete the analysis, we followed the six‐step framework by becoming familiar with the data, noting initial codes, discovering themes, reviewing themes, defining themes, and producing a report. More specifically, the first and fourth authors independently coded the qualitative survey responses line‐by‐line to generate the initial codes before grouping codes into related themes separately among the postintervention and 4‐month follow‐up data. We aimed to increase trustworthiness by considering the credibility, transferability, dependability, and confirmability of the findings (Nowell et al., 2017). We did this by keeping audit trails and reviewing the themes together to pinpoint areas of convergence and differences. Themes were then defined and are reported in the results.

## RESULTS

Results from the quantitative analyses, including the visual inspection and effect‐size estimates, are reported first. The thematic analyses of the qualitative surveys are then reported by assessment time‐point (i.e., postintervention and 4‐month follow‐up).

### Visual inspection

**Figure 1:**
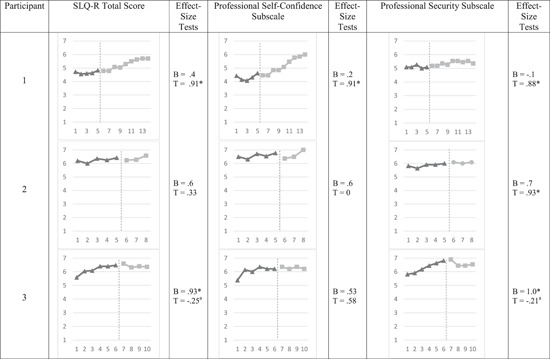
Supervisee Levels Questionnaire Scores at baseline (BL) and during video feedback (VF) supervision. The perpendicular dotted line represents the start of VF. B, baseline test; if significant, there is a BL trend before treatment, SLQ‐R, Supervisee Levels Questionnaire—Revised; T, tau‐U test; if significant, there is a treatment effect between phases. ^a^Baseline correction was accounted for in tau‐*U* test. *p < 0.05

Figure 1 presents the visual findings for each participant for the SLQ‐R total scores and the two subscales: professional self‐confidence and professional security. Details of the visual inspection for the total and subscale scores are reported below.

#### SLQ‐R total scores

Visual inspection of predictable baseline patterns revealed that participants had moderate to high baseline averages ranging from 4.56 (4 = *half of the time*) to 6.48 (6 = *most of the time*), indicating that therapist interns demonstrated a moderate to high level of therapist development during the baseline phase. Participant 1 had a baseline average of 4.67, indicating that she had the most to gain during the intervention phase. Concerning baseline trends, all participants had at least five time points which met requirements to test a baseline tend. Participants 1 and 2 had generally stable baseline trends while participant 3 demonstrated an increasing baseline trend, indicating that he had improvements in his therapist development leading up to the intervention phase. There were no overlapping data between the baseline phase and the intervention phase. In evaluating the immediate effect (e.g., comparing the last three baseline time points with the first three intervention time points), there was a small and noticeable positive intervention trend for participants 1 and 2, but a generally stable intervention trend for participant 3. Overall, participant 1 indicated a positive intervention trend across all time points. However, intervention trends were not consistent enough across the three participants to suggest a causal relation.

#### Professional Self‐Confidence subscale

Similar to the total scores, visual inspection of predictable baseline patterns revealed that participants had moderate to high baseline averages ranging from 4.07 (4 = *half of the time*) to 6.77 (7 = *always*), indicating that the therapist interns demonstrated a moderate to high level of therapist self‐confidence during the baseline phase. Most noticeably, participant 1 had a baseline average of 4.34, indicating that she had the most to gain in professional self‐confidence during the intervention phase. Concerning baseline trends, all participants had at least five time points which met requirements to test a baseline tend. Participants 1 and 2 had generally stable baseline trends while participant 3 demonstrated an increasing baseline trend, indicating that he reported improving in therapist self‐confidence during the baseline phase. There were no overlapping data between the baseline phase and the intervention phase. Evaluating the immediate effect (e.g., comparing the last three baseline time points with the first three intervention time points), there was a moderate positive intervention trend for participant 1, but a generally stable intervention trend for participants 2 and 3. Participant 1 indicated a positive intervention trend across all the time points, but this was not consistent enough across the three participants to suggest a causal relation.

#### Professional Security subscale

Similar to the total scores, visual inspection of predictable baseline patterns revealed that participants had moderate to high baseline averages ranging from 5.00 (5 = *often*) to 6.81 (7 = *always*), indicating that therapist interns reported moderate to high levels of therapist security. Participant 1 had a baseline average of 5.11, indicating that she had the most to gain in professional security from the intervention. Concerning baseline trends, all participants had at least five time points which met requirements to test a baseline tend. Participants 1 and 2 had generally stable baseline trends, while participant 3 demonstrated a noticeable increasing baseline trend, indicating that he improved in therapist security during the baseline phase. There were no overlapping data between the baseline phase and the intervention phase. Evaluating the immediate effect (e.g., comparing the last three baseline time points with the first three intervention time points), there was a small noticeable positive intervention trend for participants 1 and 2, but there was a small and noticeable negative intervention trend for participant 3. Overall, the consistent intervention trends among participant 1 and 2 may suggest a causal relation.

### Effect‐size estimates

#### SLQ‐R total scores

Baseline tests revealed that participant 3 had a significant and positive baseline trend (tau‐*U* = 0.93, SD_tau‐*U*_ = 0.35, *p* < 0.01), which confirms the visual inspection of a baseline trend we noted earlier. Participants 1 and 2 did not have a significant baseline trend (tau‐*U* = 0.4, SD_tau‐U_ = 0.41, *p* = 0.33; tau‐*U* = 0.6, SD_tau‐*U*_ = 0.41, *p* = 0.14, respectively). A significant tau‐*U* test revealed that the intervention had a positive effect on increasing participant 1's therapist development (tau‐*U* = 0.91, SD_tau‐*U*_ = 0.33, *p* < 0.01). However, there were no significant effects between the baseline and intervention phases for participants 2 and 3, even after accounting for a corrected baseline for participant 3. Consistent with the visual inspection, we cannot suggest a causal relation as there was only one significant intervention effect.

#### Professional Self‐Confidence subscale

There were no significant baseline trends for participants 1 (tau‐*U* = 0.20, SD_tau‐*U*_ = 0.41, *p* = 0.62) and 2 (tau‐*U* = 0.60, SD_tau‐*U*_ = 0.41, *p* = 0.14). Although our visual inspection indicated a potential baseline trend for participant 3, this trend did not reach significance (tau‐*U* = 0.53, SD_tau‐*U*_ = 0.36, *p* = 0.13). A significant tau‐*U* test revealed that the intervention had a positive effect on increasing participant 1's professional self‐confidence (tau‐*U* = 0.91, SD_tau‐*U*_ = 0.33, *p* < 0.01). However, there were no significant effects between the baseline and intervention phases for participants 2 and 3. Consistent with the visual inspection, we cannot suggest a causal relation with only one significant intervention effect.

#### Professional Security subscale

Baseline tests revealed that participant 3 had a significant and positive baseline trend (tau‐*U* = 1.0, SD_tau‐*U*_ = 0.36, *p* < 0.01), which confirms the visual inspection of a baseline trend we noted earlier. Participants 1 and 2 did not have a significant baseline trend (tau‐*U* = −0.1, SD_tau‐*U*_ = 0.41, *p* = 0.81; tau‐*U* = 0.7, SD_tau‐*U*_ = 0.41, *p* = 0.09, respectively). Significant tau‐*U* tests revealed that the intervention had a positive effect on improving the professional security of participant 1 (tau‐*U* = 0.88, SD_tau‐*U*_ = 0.33, *p* < 0.01) and participant 2 (tau‐*U* = 0.93, SD_tau‐*U*_ = 0.44, *p* < 0.05). However, there was not a significant effect between the baseline and intervention phases for participant 3 even after accounting for a corrected baseline. These findings suggest a causal relation, which is consistent with the visual inspection.

### Thematic analysis

We explored participants' experiences of the supervision intervention through qualitative survey responses collected at postintervention and 4‐month follow‐up. In the postintervention interview, we asked participants about their experiences of the supervision intervention. Four months after the intervention concluded, participants were asked how the use and discontinuation of VF affected their progression in learning and delivering EFT. In our thematic analysis, we identified one main theme in the post‐intervention responses and two main themes in the 4‐month follow‐up responses. This section describes the themes and subthemes, as well as relevant participant remarks.

#### Postintervention: Using VF was adventageous

The main theme in the postintervention interviews was that using VF for supervision was adventageous. Overall, participants described their experiences as positive. A metaphor from one of the participants summarized the usefulness of VF: “How could a basketball coach teach a player without watching them play or a musician improve without hearing themselves?” Three subthemes offer insight into the specific aspects of VF that therapists considered helpful.

**Return to the “here and now.”**All participants reported that they had positive experiences using VF as part of their supervision. One specific characteristic of VF that participants found helpful was to return to the “here and now” of their session, which helped participants feel as if they were back in the session again. One participant said, “VF helped focus the supervision on what I was actually doing, not merely on what I reported I was doing.” Another participant explained, “It was useful to see and hear what I said and did in therapy” and to “witness myself so I could reconsider interventions based on my struggles.”

**Identify specific strengths and areas for improvement**. VF also helped participants pinpoint specific areas of strength and areas that could be improved. One participant found that receiving “possible suggestions at specific points in the therapy sessions made the feedback more meaningful and easier to integrate into future work.” For one participant, VF helped her to see how what she did in therapy sessions “fit or did not fit with the EFT model.”

**Real‐time feedback from supervisors**. Another positive aspect of VF mentioned by participants was the usefulness of real‐time feedback offered by supervisors. VF gave participants the “ability to see yourself while your supervisor gives feedback for a rich and meaningful experience.” Participants found that the ability to watch video and view supervisors' comments simultaneously led to “clinical growth and better understanding of the feedback.”

#### Four‐month follow‐up: Discontinuing VF led to challenges

The majority of participants expressed that discontinuing VF did not affect or minimally affected their perception of the quality of supervision they received, which may have been because participants continued to receive supervision from well‐trained supervisors. However, some participants mentioned that they wished VF supervision could have continued because discontinuing it presented challenges for their clinical practice that could have been avoided through continued use of the software. Two subthemes were identified in participants' responses, lack of specific feedback and forgetting learned skills.

**Lack of specific feedback**. One participant said that discontinuing the use of VF caused a shift in her experience of supervision. VF helped her to “have exact and specific target areas where I could improve,” whereas her supervision without VF was more generalized. Another participant shared a similar experience in that VF provided her with real‐time feedback on interventions so she could know which specific interventions worked or did not work.

One participant noted that discontinuing the use of VF had a minimal impact on her perception of supervision because she continued to receive helpful live supervision from a trained EFT therapist. However, she reported, “it has been a little more difficult to integrate lessons from feedback without VF.” For one participant, discontinuing the use of VF also meant there were challenges to “having similar couple therapy experiences (e.g., structuring enactments).”

**Forgetting learned skills**. Over time, participants missed the benefit of VF. As one participant shared, “I am beginning to forget some of the skills gained due to not receiving ongoing VF supervision…I feel the use of VF really affected the quality and usefulness of supervision.” The use of VF also helped one participant to “improve [her] memory and made it easier to recall some of the feedback in future sessions.”

##### Four‐month follow‐up: Positive changes in clinical skills due to VF s

Most participants articulated positive changes to their clinical EFT skills as a result of being involved in this study and receiving VF as part of their supervision. Three subthemes were identified by participants: confidence in using EFT, strengthening EFT skills, and observation and reflection.

**Confidence in using EFT**. One participant said VF helped her become more confident in going deeper emotionally with clients and exploring heightened emotions, because “VF highlighted things that went well in sessions and increased my confidence in continuing those things.” Another participant said that she had not attempted to use EFT with clients before participating in this study. VF helped her to “move EFT from an abstract theory to a useful intervention.” Another participant also indicated that VF helped her go from feeling “novice to partially competent” in using EFT.

**Strengthening EFT skills**. In addition to building their confidence, VF also helped participants strengthen their clinical skills in using EFT. One participant said that VF “enhanced the timing of my interventions, as well as my delivery…and my ability to use key interventions (e.g., reframing, heightening) improved as a result of the study.” Participants also noted that VF was useful in helping them learn and “successfully implement EFT (e.g., evoking, enactments, exploring overt and underlying emotions)” because VF helped EFT “come to life.” Another participant said VF helped “heighten or expand experiences and show places where deeper work would be possible.” VF helped participants gain more insight into their work, thus improving their clinical EFT skills with clients. One participant stated that he had understood the EFT interventions in theory before the study; however, participating in this study helped him to implement and execute the interventions appropriately.

One participant found that VF was particularly helpful in Stage 2 of EFT because “it made me more aware of my presence in the room while also giving me clues and ideas on how to expand my work.” The same participant began to see more intense and complex couple cases after the study had finished and she expressed that it would have been helpful to continue VF supervision in her Stage 2 work with these cases.

**Observation and reflection**. One of the most useful features of VF was that it allowed therapists to watch key moments of their work in session. This enabled therapists to observe their own actions and behaviors during key segments and provided an opportunity for deep reflection on their therapeutic work. One participant found that VF helped her in that she “became more aware of [her] presence in the room, eye contact, body language, and places where [she] was stuck.” Another participant found that VF “allowed for deep reflection and processing.” Additionally, VF enabled supervisors to pinpoint times when the therapist did well, and this feedback was also affirming for therapists' learning and reflection. One participant said that using VF was analogous to “a musician listening to a recording of him or herself,” because it helped her to reflect on how well or poorly she implemented EFT interventions.

## DISCUSSION

This study investigated whether adding VF to EFT supervision improved therapists' level of development in a university training clinic focused on learning EFT. A concurrent multiple‐baseline across subjects design was used to evaluate the additional component of supervision by introducing VF to different participants at different points in time for the purpose of testing intervention effects. Participants completed quantitative surveys after each EFT session with their focal couple and responded to qualitative surveys at the end of the intervention phase and 4 months after the intervention to discern participants' perceptions of the appropriateness and their satisfaction with the supervision intervention. Overall, we found that participants demonstrated high levels of therapist development on the SLQ‐R at the start of the study, indicating the potential for a ceiling effect. Findings for the SLQ‐R total score and professional self‐confidence subscale showed an effect for one of three participants, and therefore, do not meet the minimum standards to suggest a causal relation. Two of three participants showed a significant improvement on the professional security scale, which suggests the supervision intervention had a desirable effect on professional security.

In the qualitative responses, all participants reported positive experiences of the incorporation of VF into supervision. Qualitative findings indicated that participants felt VF facilitated the development of therapeutic confidence and competence in implementing EFT interventions. They were able to witness the impact of their delivery of EFT while receiving positive and corrective feedback from their supervisor that highlighted their strengths and target areas for improvement (Palmer‐Olsen et al., 2011). This enhanced the “here and now” aspect of supervision which more traditional formats of supervision may be less capable of providing. This helped participants return to the emotional field of the moment in therapy in which supervision was needed. The participants' also conveyed that the ability to see and hear themselves while the supervisor gave feedback was important to help them gauge the quality of their delivery of EFT and to integrate specific feedback into future sessions to improve their work. Further, it helped them identify their own emotional processes in the moment, which lead to explorations of how their own emotional experiences may have affected their sessions. It also helped to identify times in the therapeutic process where they felt stuck and unsure how to navigate.

The therapists' increased ability to explore how their own emotional processes may have affected their delivery is an important finding. Therapists' own emotional processes can play a role in their ability to deliver EFT interventions like heightening emotions, evocative responses, and empathic conjectures. In EFT, heightening emotions in session occurs when therapists highlight specific responses or interactions to intensify a client's emotional experience (Johnson, 2020). If a therapist's own pain or sadness is evoked in a session, they may avoid heightening techniques to prevent their own emotions from being intensified. Working with intense emotions is generally a difficult skill to learn and teach. The fact that the supervisor is able to use VF to distill those moments in therapy where the supervisee could have heightened and intensified a client's emotional experience further and provide examples and ideas to keep clients connected to their emotional experience is a valuable tool for teaching EFT.

The 4‐month follow‐up findings showed participants experienced increased confidence in using EFT, particularly as they moved into Stage 2. This is especially meaningful as Stage 2 of EFT is the most challenging stage for therapists to learn (Bradley & Furrow, 2007; Johnson & Greenberg, 1988). Research has highlighted that effectively completing Stage 2 is related to more successful outcomes for couples (Bradley & Furrow, 2004, 2007; Dalgleish et al., 2015; Wiebe & Johnson, 2016) and therapists who struggle to execute these change events can end up in a therapeutic impasse (Bradley & Furrow, 2007; Furrow et al., 2012). After successfully completing Stage 1 (cycle de‐escalation), Stage 2 requires the restructuring of key interactions between partners. The therapist is required to “choreograph” a new dance for the couple where a previously withdrawn partner re‐engages in terms of becoming more emotionally accessible and responsive to their partner, as the more pursuing partner becomes more open and vulnerable when asking for their needs to be met (Johnson, 2020; Lee et al., 2017; McRae et al., 2014). VF software offers supervisors a tool to return therapists back to the moments in which deeper work was possible. Supervisors can then support therapists' development by role‐playing or discussing alternative techniques.

### Future directions

This article describes a proof‐of‐concept study examining the addition of VF to EFT supervision in a university training clinic (Czajkowski et al., 2015). Given the notable benefits of VF to supervising Stage 2 EFT combined with the difficulty of learning Stage 2 interventions, early findings suggest that Stage 2 EFT may offer an ideal environment for applying VF. These findings warrant future tests of the efficacy of the supervision intervention, including comparisons of various supervision formats, such as traditional video supervision versus VF supervision, to inform more effective and efficient approaches to supervision. Regarding the methodology used in this study, single‐case designs such as multiple‐baseline designs, are valuable methods with clear relevance to CFT. Multiple‐baseline designs are especially useful for proof‐of‐concept and pilot trials and for studies in which limited resources are available. The method provides opportunities for clinical research within practice settings and for doctoral dissertations, for example, where clinical research often seems out of reach. Greater adoption of these methods could help to increase meaningful research on couple and family therapy.

### Limitations

Multiple‐baseline designs work best when the dependent variable is expected to change more rapidly or when more time is allowed for the intervention phase if the dependent variable is expected to change slowly (Kratochwill et al., 2010). This study was limited by the restricted timeline imposed by the semester schedule. It is possible that a longer intervention phase would have revealed the supervision intervention had a greater effect on therapists' development. It is also expected that growth in therapist development would occur during the baseline phase since therapists were receiving supervision during that time. While it is an accepted practice in multiple‐baseline research to test whether adding a component of an intervention to an ongoing intervention improves outcomes further, it can make it difficult to document a consistent trend during the baseline phase. In this study, a consistent baseline trend was hard to achieve, and trends in the dependent variable had to be considered within this context.

The WWC standards recommend a minimum of three data points (to meet standards with reservations) to six data points (to meet standards) for each phase (Kratochwill et al., 2010). While the study was designed to meet this standard, one participant only had two data points for the intervention phase due to client cancellations. Therefore, we had to remove one participant from the quantitative analysis. While three participants are acceptable, four or more are preferable. In addition, attrition in multiple baseline designs can lead to concerns with internal validity if it is systematically related to the intervention; however, there was no indication that the attrition was related to the intervention in this study.

## CONCLUSIONS

We sought to understand whether adding VF to EFT supervision resulted in further growth in therapists' development. This proof‐of‐concept study used a concurrent multiple‐baseline across subjects design in which participants served as their own controls. In addition, qualitative responses were analyzed using thematic analysis. Findings demonstrated promise for adding VF to EFT supervision. Quantitative findings may have been affected by a ceiling effect, but the highly positive qualitative responses offer social validity data in support of VF. Future research on the efficacy of the supervision intervention component is needed to provide additional insight into the use of VF supervision.
